# The Effect of Vericiguat on Endothelial Function in Patients With Heart Failure With Reduced Ejection Fraction: A Pilot Randomized Study

**DOI:** 10.1016/j.amjcard.2026.02.062

**Published:** 2026-02-27

**Authors:** Konstantinos Sideris, Kanokwan Bunsawat, Lina Brinker, Emma Braun, Jarred Iacovelli, Arthur Chen, Jonah M. Simmons, Jia Zhao, Spencer Carter, Eleni Tseliou, Christos P. Kyriakopoulos, Thomas C. Hanff, Roberta Florido, Alison M. Brann, James C. Fang, Tom Greene, David Walter Wray, Josef Stehlik

**Affiliations:** a Division of Cardiovascular Medicine, Department of Internal Medicine, University of Utah, Salt Lake City, Utah; b Division of Cardiology, Department of Internal Medicine, George E. Wahlen Department of Veterans Affairs Medical Center, Salt Lake City, Utah; c Geriatric Research, Education, and Clinical Center, Veterans Affairs Medical Center, Salt Lake City, Utah; d Division of Geriatrics, Department of Internal Medicine, University of Utah, Salt Lake City, Utah; e Department of Nutrition and Integrative Physiology, University of Utah, Salt Lake City, Utah; f Division of Epidemiology, Department of Internal Medicine, University of Utah, Salt Lake City, Utah

**Keywords:** vericiguat, endothelial function, inflammation, quality of life, functional status, randomized control trial

## Abstract

Vericiguat is a soluble guanylate cyclase stimulator previously shown to improve cardiovascular outcomes in patients with heart failure with reduced ejection fraction (HFrEF). However, the underlying mechanisms remain incompletely understood. To evaluate the feasibility of assessing the effects of vericiguat on endothelial function, we conducted a pilot, randomized, double-blind, placebo-controlled trial of vericiguat in patients with HFrEF. Feasibility and efficacy outcomes were assessed at baseline and at 12 weeks on treatment. The primary efficacy outcome was brachial artery flow-mediated vasodilation. Secondary efficacy outcomes included N-terminal pro-brain natriuretic peptide, inflammatory markers, functional capacity, and health-related quality of life. An analysis of covariance model was used for continuous and Mantel–Haenszel tests for discretized outcomes. We enrolled 26 participants (median age 67 years, 84% male), with 25 completing 12-week follow-up, 13 in the active and 12 in the control arm. Feasibility objectives were successfully met, including enrollment, retention, drug titration, and completeness of data collection for all endpoints. In exploratory analyses, treatment with vericiguat resulted in flow-mediated dilation mean difference of 0.7%, (95% confidence interval: −1.1% to 2.5%, p = 0.40) and a nominally significant reduction of log NTproBNP of −0.42 (95% confidence interval: −0.81 to −0.04, p = 0.03). No significant differences in inflammatory biomarkers, functional capacity, or health-related quality of life were observed. We demonstrated the feasibility of assessing endothelial function in patients with HFrEF treated with vericiguat. While the pilot study size did not provide sufficient precision to confirm a treatment effect, our findings support future larger and longer studies to evaluate the therapeutic potential of soluble guanylate cyclase stimulation on endothelial function in HFrEF.

The burden of heart failure (HF) continues to increase, with approximately 6.7 million Americans over 20 years of age currently diagnosed, and its prevalence is expected to rise to 8.7 million by 2030.^1^ HF with reduced ejection fraction (HFrEF) has been associated with significant morbidity, mortality, and healthcare burden.^2^ Despite advances in pharmacologic and device-based therapies, many patients continue to experience disabling symptoms and diminished quality of life.^3^ Novel therapeutic options have been proposed to address the needs especially in patients who remain symptomatic despite optimal guideline-directed medical therapy (GDMT).^4^ In this context, the cyclic guanosine monophosphate (cGMP) pathway has emerged as a therapeutic target, particularly due to its mechanistic effects on endothelial function, nitric oxide (NO) signaling, and inflammation.^5^

Vericiguat, an oral soluble guanylate cyclase (sGC) stimulator, enhances cGMP production independently of NO.^6^ In the VICTORIA (VerICiguaT Global Study in Subjects with Heart Failure with Reduced Ejection Fraction) trial, vericiguat reduced the risk of cardiovascular death or HF hospitalization in patients with HFrEF, yet the precise mechanisms underlying this benefit remain uncertain.^7^ Further analyses from the VICTORIA trial suggested no significant changes in left ventricular ejection fraction (LVEF), while in earlier phase studies, higher-dose vericiguat was associated with a modest improvement in LVEF without corresponding changes in left ventricular volumes.^8^ These data raised the hypothesis that vericiguat’s therapeutic effects may be mediated through an improvement in endothelial function rather than direct myocardial effects,^9^ yet this important question has not been evaluated in the clinical setting.

Endothelial dysfunction is a hallmark of HFrEF and is linked to impaired vasodilation, chronic inflammation, reduced exercise tolerance, and worse health-related quality of life (hrQOL).^10–13^ Flow-mediated dilation (FMD), a noninvasive measure of endothelial function, is often impaired in HFrEF, even in patients receiving optimized GDMT.^10,12,14,15^ Emerging data suggest that endothelial dysfunction may be a modifiable contributor to poor outcomes in HFrEF, and therapies targeting vascular health may offer substantial clinical benefits.^16,17^

In this pilot randomized, placebo-controlled study, we evaluated the feasibility of assessing the effect of vericiguat on endothelial function, inflammation, physical capacity, and patient-reported health status over 12 weeks of treatment in patients with HFrEF. Exploratory analysis included the treatment effect of vericiguat in endothelial function, inflammatory biomarker levels, and functional and hrQOL measures. This study is one of the first efforts to directly examine the vascular and inflammatory effects of vericiguat in a HFrEF population.

## Methods

### Trial design and ethical approval

This was a single-center, randomized, double-blind, placebo-controlled trial conducted at the University of Utah and the Salt Lake City Veterans Affairs Medical Center. The study protocol was approved by the Institutional Review Boards (IRB) of both participating institutions (IRB: 00152530). All aspects of the study conformed to the standards set by the Declaration of Helsinki, except for registration in a database. All experimental procedures were explained to participants in writing and verbally, and written informed consent was obtained from all participants before study participation. The trial was investigator-initiated and supported by Merck Investigator Study Program and the University of Utah. The investigators were responsible for the study design, data collection, data analysis, and interpretation of results. Statistical analyses were performed by the study statisticians and independently reviewed by the study team.

### Study population

Eligible participants were adults (≥18 years) with HFrEF and New York Heart Association class II or III symptoms. Further inclusion criteria included a LVEF ≤ 45% within the past 12 months, systolic blood pressure ≥90 mm Hg, and optimized use of GDMT. The full list of inclusion and exclusion criteria is provided in Supplemental Methods.

### Randomization and treatment

Following the screening visit, eligible participants were randomly assigned in a 1:1 ratio to receive either vericiguat or matching placebo for a total of 12 weeks. Randomization was stratified by participating site, used randomly permuted blocks, and was performed by a computer-generated randomization software managed by the study investigational pharmacy. Study drug was administered once daily, starting at 2.5 mg, increased to 5 mg at week 2, and to the target dose of 10 mg at week 4 as tolerated, guided by blood pressure (BP) and clinical symptoms. To enhance the likelihood of achieving and maintaining the target dose of 10 mg, investigators were encouraged to address dosing at each visit according to the patient’s BP and symptomatic status. Patients were evaluated for study-related adverse events at weeks 2, 4, 8, and 12. Throughout the study, participants remained on GDMT for HFrEF. Both participants and study personnel were blinded to treatment allocation throughout the trial. Study drug dispensing and compliance monitoring were coordinated through the investigational pharmacy.

### Trial conduct

All data collection took place with patients in the supine position in a thermoneutral environment. All patients reported to the laboratory at least 4 hours postprandial, having taken their medication with food or water according to their usual routine, and abstained from caffeine, alcohol, and exercise for 24 hours prior. Data collection included anthropometrics, venous blood samples, BP, endothelial function (brachial artery FMD), functional capacity via the six-minute walk test (6MWT), and hrQOL assessment via standardized questionnaires (Kansas City Cardiomyopathy Questionnaire-12 [KCCQ-12], visual analog scale [VAS]). To minimize physiologic interference between assessments, FMD was performed first, followed by blood sample collection, and subsequently by the 6MWT. Venous blood samples were processed using standard clinical procedures at the Salt Lake City Veterans Affairs Medical Center and at the University of Utah Hospital. Follow-up testing occurred while participants were receiving study drug and at the same time of day for baseline and 12-week visits; assessments were not timed to a fixed interval after the last received dose.

### Brachial artery FMD measurements and analysis

FMD testing for the assessment of conduit artery endothelial function was performed in accordance with established guidelines.^18^ A detailed description of the FMD assessment protocol is provided in Supplemental Methods.

### Blood biomarker assays

Blood samples were centrifuged to collect plasma and serum, and either directly analyzed or stored at −80°C until analysis. A detailed description of the biomarkers assessment protocol is provided in Supplemental Methods.

### Six-minute walk test

The 6MWT was conducted following a standardized protocol.^19^ Patients were instructed to cover the maximum distance possible in 6 min, at a self-selected walking speed, and were unaccompanied and without verbal encouragement, to avoid any social influence on the walking speed. The 6MWT distance is used to estimate functional capacity and is highly reproducible in patients with HFrEF.^19^

### hrQOL instruments

Both disease-specific and generic patient-reported outcome metrics were used. HF-specific health status was assessed with the KCCQ-12. The KCCQ-12 has been shown be a valid, reliable, and sensitive measure in a variety of causes of HF.^20^ Generic health status was assessed with a VAS.^21^

### Outcomes

The primary outcome was endothelium-dependent vascular function at 12 weeks, measured by brachial artery FMD. Secondary outcomes included circulating biomarkers, functional capacity, and hrQOL at 12 weeks. Circulating biomarkers of inflammation and cardiac stress included high-sensitivity C-reactive protein (hsCRP), tumor necrosis factor-*α* (TNF-*α*), interleukin (IL)-6, IL-18, and N-terminal pro-brain natriuretic peptide (NT-proBNP). Functional capacity was assessed via the 6MWT. HrQOL was measured using the KCCQ-12 overall summary score and a VAS.

### Statistical analysis

Patient baseline characteristics were summarized by assignment group (vericiguat and placebo). Standard summary descriptions were used, including frequencies, percentages, and medians. Measures of variation were presented as the median (interquartile range). All analyses were conducted on an intention-to-treat basis. For each continuous variable (FMD, 6MWT, KCCQ, VAS, log hsCRP, log NT-proBNP, and log IL-18), an analysis of covariance model was used, modelling the 12-week measurement as the dependent variable and the vericiguat treatment as the treatment variable, while adjusting for the baseline measurement. The biomarkers hsCRP, NT-proBNP, and IL-18 were log-transformed to address skewness. The biomarkers TNF-*α* and IL-6 were discretized into binary variables due to high rates of values below the detection limit of the assay. Cutoff values were defined according to clinically meaningful thresholds established in prior studies.^22–24^ For these discretized biomarkers TNF-*α* and IL-6, we used Mantel–Haenszel tests to assess the association between treatment and biomarker detectability at 12 weeks, stratified by baseline detectability status. As the purpose of this trial was to evaluate feasibility, the sample size was not sufficient to determine treatment effect. Accordingly, we provide point estimates and 95% confidence intervals (CI) for all treatment comparisons on outcomes, and report 2-sided p values, but interpret analyses of treatment effects as exploratory. All computations were conducted using R version 4.4.2 (R Core Team 2024).

## Results

### Study participants

A total of 26 participants were enrolled at 2 sites from May 25, 2023, through July 18, 2024, and follow-up was completed on October 10, 2024. Twenty-five (96%) participants completed the 12-week follow-up, with 1 participant in the placebo group lost to follow-up after 25 days due to relocation.

Baseline characteristics of the patients are shown in Table 1. The median age of enrolled patients was 67 (57, 73) years, 84% were male, and 96% were White. At randomization, 76% of participants were classified as New York Heart Association Class II, and had a median LVEF of 33 (27, 39) %. Most patients were receiving comprehensive GDMT, including beta-blockers (100%), angiotensin converting enzyme inhibitor/angiotensin receptor blocker/angiotensin receptor/neprilysin inhibitor (88%), mineralocorticoid receptor antagonists (92%), and sodium-glucose cotransporter-2 inhibitors (60%). Diuretics were prescribed to 88% of participants, the majority of whom (59%) received a furosemide equivalent daily dose of 40 mg (Table 1).

### Study feasibility and drug titration

Feasibility goals were achieved, including timely enrollment, 100% successful randomization, and high data completeness for all outcomes. All randomized participants received at least one dose of study drug, and by week 4, the target dose of 10 mg once daily was achieved in 84% of participants overall, including 92% in the vericiguat group and 75% in the placebo group. At week 12, 91% of participants remained on the 10 mg dose (100% placebo, 83% vericiguat) (Table 2). Adherence to study procedures was high, with complete primary outcome data collected in 96% (*n* = 25) of randomized participants. Complete biomarker assessments were available for >98% of study visits. Functional status and hrQOL were evaluated for all randomized participants who completed the study (*n* = 25, 96%).

### Primary outcome

**Figure 1.**
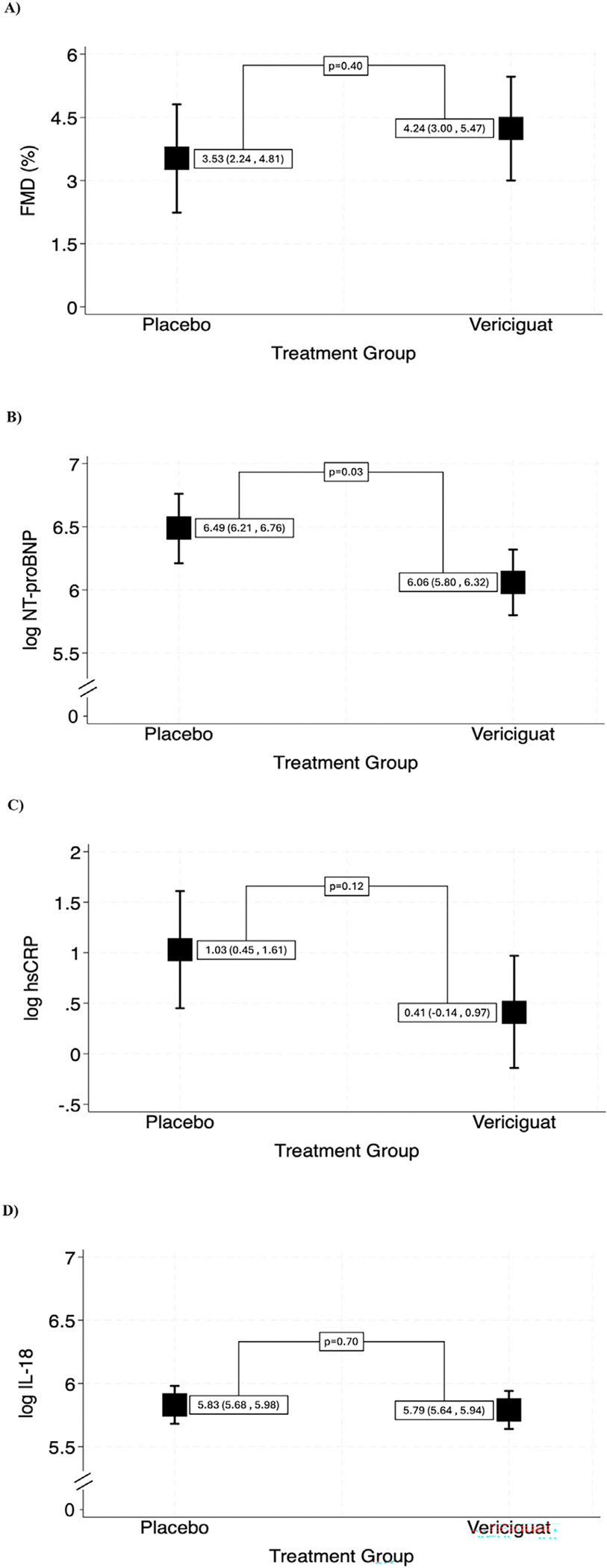

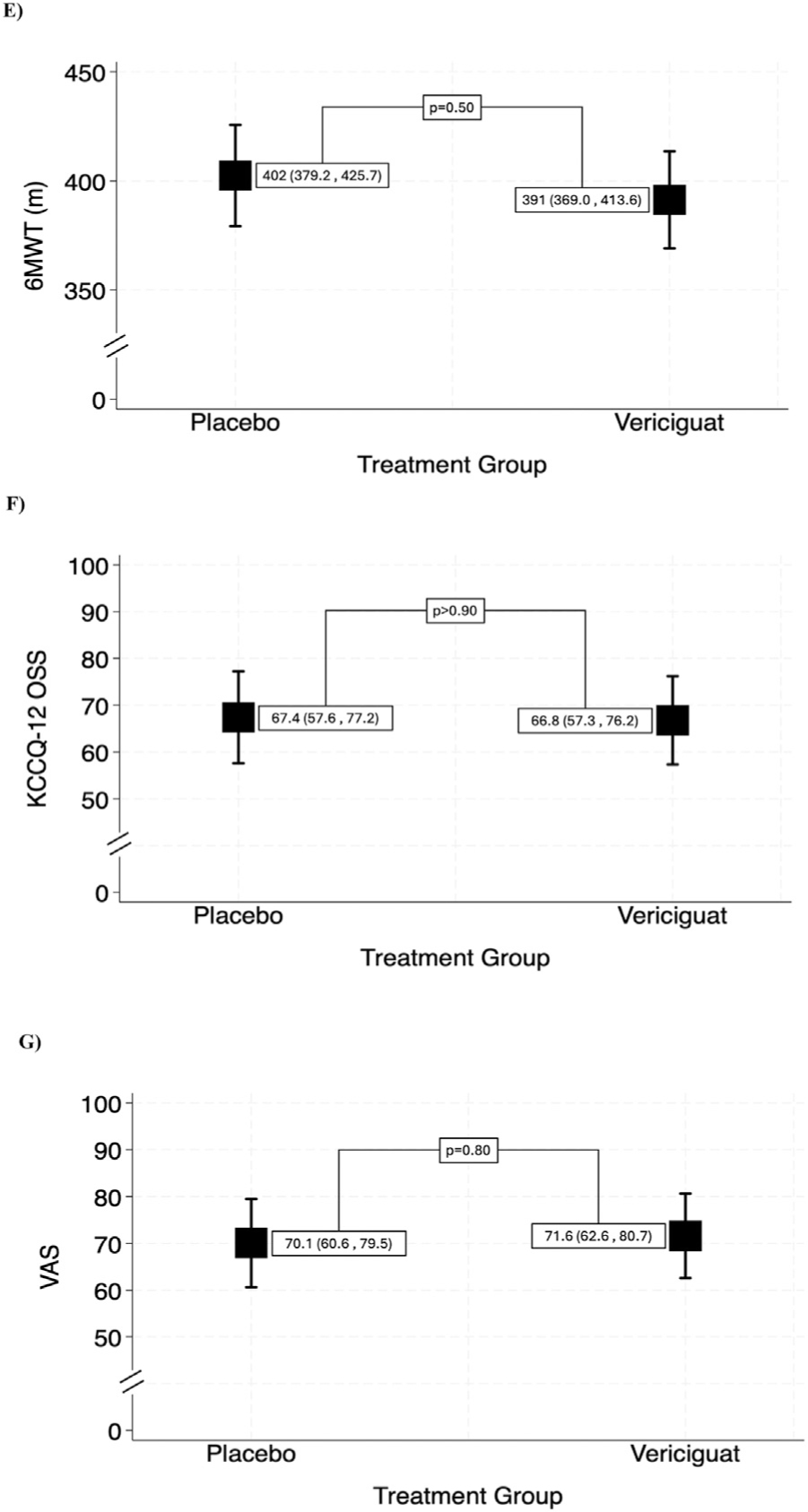
Adjusted mean of study outcomes at 12 weeks with 95% confidence intervals (CI) stratified by treatment group: (A) flow-mediated dilation (FMD), (B) N-terminal pro-brain natriuretic peptide (NT-proBNP), (C) high-sensitivity C-reactive protein (hsCRP), (D) interleukin 18 (IL-18), (E) six-minute walk test (6MWT), (F) Kansas City Cardiomyopathy Questionnaire-12 overall summary score (KCCQ-12 OSS), (G) visual analog scale (VAS).

FMD was assessed in 26 patients at baseline and 25 at week 12 due to loss to follow-up of 1 participant in the placebo group. Mean FMD increased from 3.6% at baseline to 4.2% at 12 weeks in the vericiguat group but decreased from 3.7% at baseline to 3.6% at 12 weeks in the placebo group (Table 3, Supplementary Figure 1). At week 12, the adjusted mean FMD was 4.2% (95% CI: 3.00 to 5.47) in the vericiguat group versus 3.5% (95% CI: 2.24 to 4.81) in the placebo group (estimated treatment effect [ETE]:0.71, 95% CI: −1.1 to 2.5; p = 0.40) (Figure 1). Baseline and 12-week measurements of baseline brachial artery diameter, peak brachial artery diameter, shear rate area under the curve, FMD/shear rate area under the curve, and BP are shown in Supplementary Table.

## Secondary Outcomes

### Circulating biomarkers

NT-proBNP levels were assessed in 26 patients at baseline and 25 at week 12 due to loss to follow-up of 1 participant in the placebo group. Treatment with vericiguat resulted in a significant reduction of NT-proBNP. Mean NT-proBNP decreased from 1,306 pg/ml at baseline to 1,231 pg/ml at 12 weeks in the vericiguat group but increased from 623 pg/ml at baseline to 659 pg/ml at 12 weeks in the placebo group (Table 3, Supplementary Figure 1). The adjusted mean log-transformed NT-proBNP at week 12 was 6.06 (95% CI: 5.80 to 6.32) in the vericiguat group compared to 6.49 (95% CI: 6.21 to 6.76) in the placebo group (ETE: −0.42, 95% CI: −0.81 to −0.04; p = 0.03) (Figure 1). hsCRP levels were assessed in 26 patients at baseline and 25 at week 12 due to loss to follow-up of 1 participant in the placebo group. hsCRP values showed a trend favoring vericiguat treatment. hsCRP mean values decreased from 2.90 mg/L at baseline to 2.33 mg/L at 12 weeks in the vericiguat group and increased from 2.93 pm/L at baseline to 4.22 mg/L at 12 weeks in the placebo group (Table 2, Supplementary Figure 1). The adjusted mean log-transformed hsCRP at week 12 was 0.41 (95% CI: −0.14 to 0.97) in the Vericiguat group compared to 1.03 (95% CI: 0.45 to 1.61) in the placebo group (ETE: −0.62, 95% CI: −1.4 to 0.18; p = 0.12) (Figure 1).

IL-18 levels were assessed in 26 patients at baseline and 25 at week 12 due to loss to follow-up of 1 participant in the placebo group. Mean IL-18 levels decreased slightly over 12 weeks, from 477 to 461 pg/ml in the vericiguat group and from 339 to 334 pg/ml in the placebo group (Table 3, Supplementary Figure 1). The adjusted mean log-transformed IL-18 at week 12 was 5.79 (95% CI: 5.64 to 5.94) in the vericiguat group compared to 5.83 (95% CI: 5.68 to 5.98) in the placebo group (ETE: −0.04, 95% CI: −0.26 to 0.18; p = 0.70) (Figure 1).

**Figure 2.**
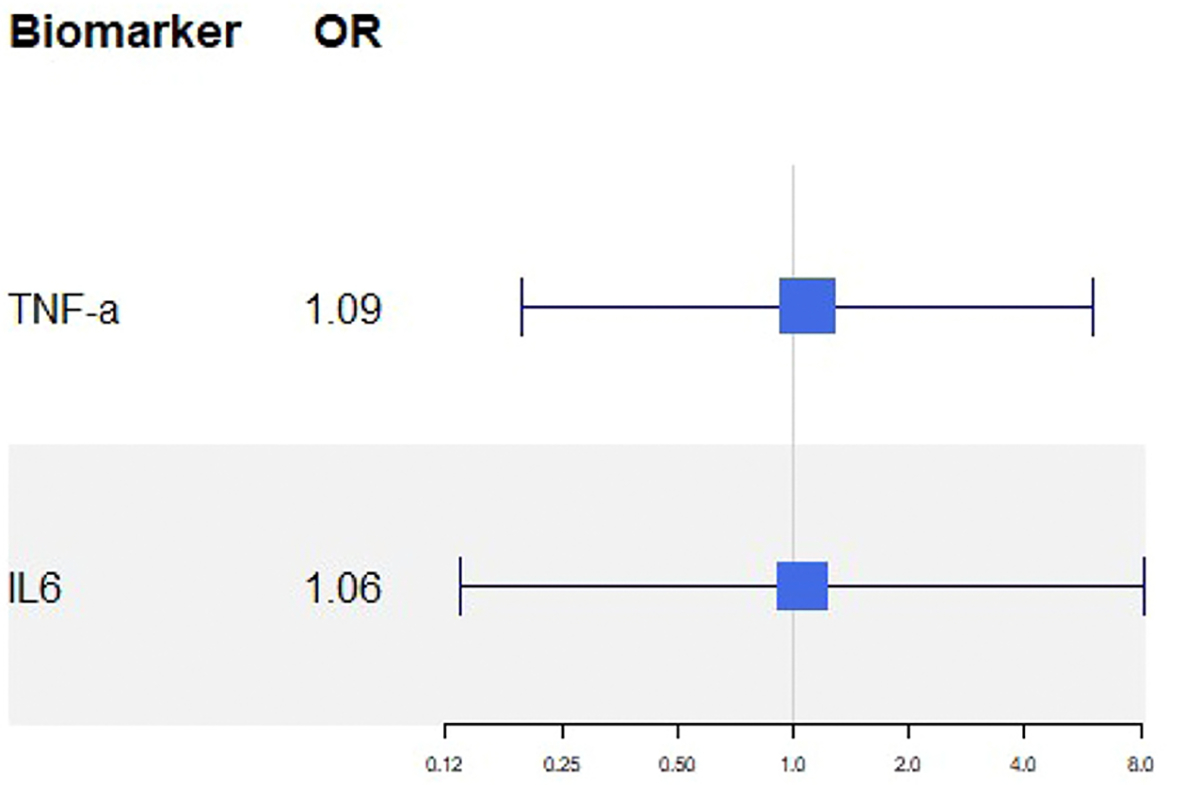
Common odds ratio for tumor necrosis factor-*α* (TNF-*α*) and interleukin-6 (IL-6) adjusted for baseline.

IL-6 levels were assessed in 26 patients at baseline and 24 at week 12. One patient in the placebo group was lost to follow-up, and one sample in the vericiguat group could not be processed due to sample quality. At baseline, 4 (31%) participants in the vericiguat group and 1 (8.3%) participant in the placebo group had IL-6 levels ≥2.0 pg/ml. By week 12, this proportion remained stable in the vericiguat group with 4 (33%) patients but increased in the placebo group to 3 (25%) patients (Table 3, Supplementary Figure 1). The common odds ratio for IL-6 was 1.06 (95% CI: 0.14 to 8.13; p = 0.96) (Figure 2).

TNF-*α* levels were assessed in 26 patients at baseline and 24 at week 12. One patient in the placebo group was lost to follow-up, and one sample in the vericiguat group could not be processed due to sample quality. A similar pattern was observed for TNF-*α* levels. At baseline, 7 (54%) participants in the vericiguat group and 4 (33%) participants in the placebo group had TNF-*α* levels ≥1.7 pg/ml. By week 12, this proportion decreased in the vericiguat group with 4 (31%) patients, but remained stable in the placebo group to 4 (33%) patients (Table 3, Supplementary Figure 1). The common odds ratio for TNF-*α* was 1.09 (95% CI: 0.20 to 6.04; p = 0.92), showing no significant between-group difference (Figure 2).

### Functional capacity and hrQOL

Functional capacity, as assessed by the 6MWT, was evaluated in 26 patients at baseline and 25 at week 12 due to loss to follow-up of 1 participant in the placebo group. 6MWT distance did not significantly change over the 12-week period in either group. In the vericiguat group, the mean 6MWT distance was 390 meters at baseline and 388 meters at 12 weeks. In the placebo group, the mean 6MWT distance was 398 meters at baseline and 406 meters at 12 weeks (Table 3, Supplementary Figure 1). At 12 weeks, the adjusted mean 6MWT distance was 391 meters (95% CI: 369.0 to 413.6) in the vericiguat group and 402 meters (95% CI: 379.2 to 425.7) in the placebo group, yielding an ETE of −11 meters (95% CI: −43 to 21; p = 0.50) (Figure 1).

Patient-reported hrQOL status was assessed in 26 patients at baseline and 25 at week 12 due to loss to follow-up of 1 participant in the placebo group. Both study groups showed modest improvement in hrQOL over the 12-week period. The mean KCCQ-12 overall summary score increased from 63 to 68 in the vericiguat group and from 59 to 66 in the placebo group (Table 3, Supplementary Figure 1). At week 12, adjusted mean KCCQ-12 scores were 66.8 (95% CI: 57.3 to 76.2) in the vericiguat group and 67.4 (95% CI: 57.6 to 77.2) in the placebo group (ETE: −0.64, 95% CI: −14 to 13; p > 0.90) (Figure 1). Similarly, mean VAS scores increased from 55 to 70 in the vericiguat group and from 66 to 72 in the placebo group (Table 3, Supplementary Figure 1). The adjusted mean VAS score at 12 weeks was 71.6 (95% CI: 62.6 to 80.7) in the Vericiguat group and 70.1 (95% CI: 60.6 to 79.5) in the placebo group (ETE:1.5, 95% CI: −12 to 15; p = 0.80) (Figure 1).

### Adverse events and safety

The study drug was generally well-tolerated, with no unexpected safety concerns observed during the 12-week treatment period. No deaths, life-threatening events, or persistent disabilities were reported in either group. All-cause hospitalizations occurred in 4 (16%) participants overall: 3 (23%) in the vericiguat group and 1 (8.3%) in the placebo group (p = 0.60).

Detailed information on adverse events is listed in Table 4. Adverse events were generally mild and similar between groups. The most common events included reported dizziness (44% overall; 50% placebo vs 38% vericiguat, p = 0.60), fatigue (24% overall; 25% placebo vs 23% vericiguat, p > 0.90), and hypotension (8% overall; 8% placebo vs 8% vericiguat, p > 0.90). Headache was reported in 1 (8%) participant in the placebo group and none in the vericiguat group. New-onset anemia occurred in 1 (8%) vericiguat-treated participant (Table 4).

## Discussion

In this pilot, randomized trial, we demonstrated the feasibility of conducting a 12-week, placebo-controlled study assessing the effect of vericiguat on vascular function, inflammation, functional capacity, and hrQOL status in patients with HFrEF. Feasibility objectives were successfully met, including timely enrollment, high rates of retention, drug titration, and completeness of data collection for all endpoints. While the directions of the treatment comparisons of some study outcomes were consistent with a possible treatment benefit, this pilot trial was not designed to evaluate the hypothesis of treatment benefit. This needs to be evaluated in appropriately powered efficacy trials.

In the VICTORIA trial, the pivotal clinical trial of vericiguat in patients with HFrEF and recent worsening HF and higher disease severity, treatment with vericiguat led to a lower rate of HF hospitalizations compared to placebo.^7^ In contrast, our pilot study examined a clinically stable, ambulatory cohort with high utilization of contemporary GDMT, more closely resembling populations studied in VICTOR (Vericiguat in patients with chronic HF and reduced ejection fraction).^25^ The mechanistic pathways through which vericiguat confers clinical benefit in HFrEF remain incompletely understood, and the patient populations most likely to derive vascular benefit from sGC stimulation have not been clearly defined. Differences in disease severity, inflammatory burden, neurohormonal activation, and baseline endothelial dysfunction may influence vascular responsiveness and could account for heterogeneity in observed physiological effects across studies. These observations highlight the need for future mechanistic studies designed to identify target populations in which sGC stimulation yields the greatest vascular and clinical benefit. Our pilot supports this by providing data on the feasibility of direct endothelial function assessment in the HFrEF population.

Endothelial dysfunction is a hallmark of HFrEF and is recognized and a potentially modifiable contributor to disease progression.^26^ FMD is often impaired in patients with HFrEF despite optimized GDMT.^10,12,14,15,27^ In individuals without HF or overt cardiovascular disease, brachial artery FMD is typically in the range of approximately 6.5% to 7%, with lower values reflecting endothelial dysfunction.^28,29^ In our study, baseline FMD was low in both groups (3.61% in the vericiguat group vs 3.66% in the placebo group), despite high use of vasoactive agents including renin-angiotensin-aldosterone inhibitors (93% in the vericiguat group vs 83% in the placebo group). Preclinical data offer compelling mechanistic support for the hypothesis that vericiguat’s benefits might be mediated through vascular effects. In a rat model of HF, the sGC stimulator ataciguat improved endothelial function, restored vascular sensitivity to NO, and reduced platelet activation.^30^ This pilot study demonstrated the feasibility of assessing endothelial function via FMD in a randomized trial setting, with high protocol adherence and successful data collection. Exploratory analyses indicated a modest increase in FMD over 12 weeks, whereas FMD slightly declined in the placebo group. The mean difference in FMD between vericiguat and placebo was 0.7%. This may suggest important clinical implications, as prior data suggest a 1% increase in FMD is associated with 13% reduction in future cardiovascular risk.^17^ These results could provide preliminary data to guide the design and power calculations for future trials evaluating the effect of sGC stimulation on endothelial function.

Strong preclinical evidence also supports a role for the NO–sGC–cGMP axis in modulating NLRP3 inflammasome activity, which is increasingly recognized as a driver of HF-related inflammation.^31^ Recently, vericiguat has been shown to reduce hsCRP and oxidative stress markers in patients with HFrEF, suggesting systemic anti-inflammatory potential.^32^ We demonstrated the feasibility of collecting and evaluating serial inflammatory biomarker data in a HFrEF population over 12 weeks of vericiguat treatment. While this pilot study was not designed or powered to assess the anti-inflammatory effects of vericiguat, our exploratory analyses results were consistent with previous data, showing a nominal effect toward reduction of inflammatory markers, including hsCRP, IL-6, IL-18, and TNF-*α* after 12 weeks of treatment with vericiguat. The strong association between endothelial function and inflammatory biomarkers seen in other disease states suggests that favorable vascular effects of vericiguat may be more pronounced in individuals with greater reductions in inflammation.^33^ Future studies should explore these mechanisms using longer treatment durations and enriched populations with heightened inflammatory activity to better characterize the immunomodulatory potential of vericiguat.

Consistent with inflammatory biomarkers, our pilot study showed the feasibility of measuring NT-proBNP levels, a biomarker of myocardial wall stress and a potent prognostic marker in HFrEF.^34^ While the reduction in NT-proBNP observed in the vericiguat group was statistically significant, these findings must be interpreted cautiously given the small sample size and exploratory nature of the trial. This reduction of NT-proBNP is consistent with findings in prior trials and adds to the accumulating evidence that sGC stimulation may attenuate cardiac stress independent of direct myocardial remodeling.^35^ However, in an earlier phase study, vericiguat did not significantly reduce NT-proBNP at 12 weeks in the primary pooled analysis, although a dose–response relationship was observed at higher doses. Differences in study design and patient population may explain this discrepancy, as patients were enrolled early after a worsening HF event in a dose-finding framework, whereas our pilot study evaluated sustained target-dose therapy in clinically stable patients.^8^

Key feasibility objectives were confirmed in collecting functional and patient-reported outcome measures, including the 6MWT, KCCQ-12, and VAS. As anticipated, the exploratory analysis demonstrated modest, nonsignificant changes in functional capacity and hrQOL between treatment arms. These exploratory findings are consistent with data showing no significant differences in 6MWT between vericiguat and placebo groups in other clinical trials, underscoring the limited effect of vericiguat on functional capacity.^36^ Similarly, results from this pilot study support findings from the VICTORIA trial in which vericiguat significantly reduced adverse outcomes but led to only modest and comparable improvements in KCCQ scores across treatment arms.^7,37^ The clinical benefit of HFrEF therapies has been shown to be largely independent of baseline hrQOL, suggesting that symptomatic improvement may lag behind physiological changes or require longer treatment duration.^38^

Our study has several limitations. The sample size was small, limiting our power to evaluate the treatment effect of vericiguat in vascular, clinical, or biomarker outcomes. The trial duration was relatively short, and it is possible that a longer follow-up period is required to observe the full physiological and symptomatic effects of sGC stimulation. The study population was predominantly White and male, limiting generalizability to more diverse HF populations. Although participants were maintained on stable GDMT whenever possible, there was one initiation of a new GDMT medication and 18 dose adjustments (8 downtitrations and 10 uptitrations) based on clinical indication during the 12-week study period, which could have influenced study outcomes independent of study treatment. In addition, patient-level factors known to modulate endothelial function, including smoking status, underlying atherosclerotic burden, and arterial stiffness, were not systematically characterized in this pilot trial and may further modify treatment response. Finally, while FMD is a validated surrogate of endothelial function, it is operator-dependent and may be subject to technical variability. However, adherence to FMD guidelines improves reproducibility.^39^

In conclusion, this pilot randomized trial established the feasibility of evaluating endothelial function in patients with HFrEF treated with vericiguat and confirmed endothelial function impairment despite GDMT. We also successfully collected data on inflammation, functional capacity, and hrQOL. While the pilot study size did not provide sufficient precision to confirm a treatment effect, our findings support future studies to evaluate the therapeutic potential of sGC stimulation on endothelial function in HFrEF.

## Supplementary Material

Suppl Material

Supplementary material associated with this article can be found in the online version at https://doi.org/10.1016/j.amjcard.2026.02.062.

## Figures and Tables

**Table 1:** Characteristics of the patients at baseline

Characteristic	Overall *N* = 25*	Placebo *N* = 12*	Vericiguat *N* = 13*

Age (years)	67 (57, 73)	67 (59, 71)	68 (52, 79)
Sex			
Female	4 (16%)	1 (8.3%)	3 (23%)
Male	21 (84%)	11 (92%)	10 (77%)
Ethnicity			
Not Hispanic or Latino	24 (96%)	12 (100%)	12 (92%)
Hispanic or Latino	1 (4.0%)	0 (0%)	1 (7.7%)
Race			
White	24 (96%)	12 (100%)	12 (92%)
Native Hawaiian or Other Pacific Islander	1 (4.0%)	0 (0%)	1 (7.7%)
Body mass index (kg/m^2^)	30.8 (28.8, 34.5)	30.9 (29.6, 34.0)	29.3 (26.7, 34.5)
Systolic blood pressure (mm Hg)	109 (97, 124)	99 (91, 122)	112 (106, 126)
Diastolic blood pressure (mm Hg)	71 (63, 76)	70 (62, 75)	75 (67, 79)
Heart rate (bpm)	65 (59, 70)	66 (61, 74)	64 (52, 70)
Comorbidities			
Ischemic cardiomyopathy	9 (36%)	5 (42%)	4 (31%)
NYHA class			
II	19 (76%)	8 (67%)	11 (85%)
III	6 (24%)	4 (33%)	2 (15%)
Hypertension	18 (72%)	7 (58%)	11 (85%)
Atrial fibrillation	12 (48%)	4 (33%)	8 (62%)
Type 2 diabetes mellitus	14 (56%)	8 (67%)	6 (46%)
Hyperlipidemia	14 (56%)	8 (67%)	6 (46%)
Coronary artery disease	12 (48%)	7 (58%)	5 (38%)
Peripheral vascular disease	2 (8.0%)	1 (8.3%)	1 (7.7%)
Echocardiography parameters			
LVEF (%)	33 (27, 39)	27 (24, 39)	36 (33, 39)
LVIDd (cm)	6.10 (5.50, 6.40)	6.10 (5.50, 6.30)	6.10 (5.50, 6.60)
Laboratories			
Hemoglobin (g/dL)	15.20 (13.80, 16.20)	15.25 (14.00, 16.20)	14.50 (13.50, 16.80)
Hematocrit (%)	45.3 (41.6, 48.0)	46.7 (42.2, 48.8)	44.0 (41.6, 47.2)
White blood cells (10^9^/L)	7.10 (6.23, 8.17)	6.48 (6.20, 7.90)	7.75 (6.49, 8.22)
Sodium (mEq/L)	139.00 (138.00, 140.00)	139.00 (138.00, 140.00)	139.00 (138.00, 141.00)
Potassium (mEq/L)	4.40 (4.30, 4.50)	4.40 (4.30, 4.50)	4.40 (4.30, 4.50)
Creatinine (mg/dL)	1.14 (1.04, 1.44)	1.13 (0.99, 1.34)	1.14 (1.06, 1.64)
Blood urea nitrogen (mg/dL)	21.0 (18.0, 28.0)	20.0 (16.5, 27.5)	22.0 (20.0, 29.0)
Glucose (mg/dL)	100 (88, 135)	118 (88, 133)	98 (88, 135)
Concomitant medications			
Beta blockers	25 (100%)	12 (100%)	13 (100%)
ACEI/ARB/ARNI	22 (88%)	10 (83%)	12 (92%)
MRA	23 (92%)	12 (100%)	11 (85%)
SGLT2i	15 (60%)	8 (67%)	7 (54%)
Diuretics	22 (88%)	11 (92%)	11 (85%)
Furosemide daily dose (mg)			
20	4 (18%)	1 (9.1%)	3 (27%)
40	13 (59%)	7 (64%)	6 (55%)
80	5 (23%)	3 (27%)	2 (18%)

ACEI = angiotensin converting enzyme inhibitor; ARB = angiotensin receptor blocker; ARNI = angiotensin receptor/neprilysin inhibitor; LVEF = left ventricular ejection fraction; LVIDd = left ventricular internal diameter at end-diastole; MRA = mineralocorticoid receptor agonist; NYHA = New York Heart Association; SGLT2i = sodium-glucose cotransporter-2 inhibitor.

* Median (Q1, Q3); *n* (%).

**Table 2:** Vericiguat and placebo dosing achieved at weeks 4 and 12 follow-up visits by treatment group

	Overall *N* = 25*	Placebo *N* = 12*	Vericiguat *N* = 13*

Dose at week 4			
2.5 mg	0 (0%)	0 (0%)	0 (0%)
5 mg	4 (16%)	3 (25%)	1 (7.7%)
10 mg	21 (84%)	9 (75%)	12 (92%)
Dose at week 12			
2.5 mg	1 (4.3%)	0 (0%)	1 (8.3%)
5 mg	1 (4.3%)	0 (0%)	1 (8.3%)
10 mg	21 (91%)	11 (100%)	10 (83%)

* *n* (%).

**Table 3:** Descriptive summary of study outcomes

	Placebo		Vericiguat	
Outcome	Baseline *N* = 12*	Week 12 *N* = 12*	Baseline *N* = 13*	Week12 *N* = 13*

FMD (%)	3.66 (1.33)	3.55 (2.72)	3.61 (2.36)	4.22 (2.33)
NT-proBNP (pg/ml)	623 (342)	659 (482)	1,306 (1,175)	1,231 (1,290)
hsCRP (mg/L)	2.93 (2.57)	4.22 (4.28)	2.90 (1.87)	2.33 (1.77)
IL-18 (pg/ml)	339 (193)	334 (205)	477 (275)	461 (312)
IL-6 (pg/ml)				
<2.0	11 (92%)	9 (75%)	9 (69%)	8 (62%)
≥2.0	1 (8.3%)	3 (25%)	4 (31%)	4 (31%)
TNF-*α* (pg/ml)				
<1.7	8 (67%)	8 (67%)	6 (46%)	8 (62%)
≥1.7	4 (33%)	4 (33%)	7 (54%)	4 (31%)
6MWT (m)	398 (103)	406 (97)	390 (155)	388 (148)
KCCQ-12 OSS (0–100)	59 (20)	66 (23)	63 (22)	68 (24)
VAS (0–100)	66 (21)	72 (13)	55 (23)	70 (20)

6MWT = six-minute walk test; FMD = flow-mediated dilation; hsCRP = high-sensitivity C-reactive protein; IL = interleukin; KCCQ-12 OSS = Kansas City Cardiomyopathy Questionnaire-12 overall summary score; NT-proBNP = N-terminal pro-brain natriuretic peptide; TNF-*α* = tumor necrosis factor-*α*; VAS = visual analog scale.

* Mean (SD); *n* (%).

**Table 4:** Adverse events during study period stratified by study group

Characteristic	Overall *N* = 25^†^	Placebo *N* = 12^†^	Vericiguat *N* = 13^†^	p value^‡^

Serious adverse events				
Death	0 (0%)	0 (0%)	0 (0%)	>0.90
Life-threatening	0 (0%)	0 (0%)	0 (0%)	>0.90
All-cause hospitalization	4 (16%)	1 (8.3%)	3 (23%)	0.60
Adverse events				
Hypotension	2 (8%)	1 (8%)	1 (8%)	>0.90
Headache	1 (4%)	1 (8%)	0 (0%)	0.50
Dizziness	11 (44%)	6 (50%)	5 (38%)	0.60
Fatigue	6 (24%)	3 (25%)	3 (23%)	>0.90
Cough	0 (0%)	0 (0%)	0 (0%)	>0.90
New onset anemia	1 (4%)	0 (0%)	1 (8%)	>0.90

† n (%) of patients that experienced at least one adverse event or serious adverse event during the study period. Some patients may have experienced a type of adverse event more than once.

‡ Fisher’s exact test; Pearson’s Chi-squared test.
