# Supplementary material for: The Effect of Vericiguat on Endothelial Function in Patients With Heart Failure With Reduced Ejection Fraction: A Pilot Randomized Study
Source: Am J Cardiol. Author manuscript; Available in PMC 2026 Apr 24. (PMC13107246; doi:10.1016/j.amjcard.2026.02.062)
Supplement: Suppl Material [file NIHMS2162098-supplement-Suppl_Material.pdf]

SUPPLEMENTAL MATERIAL

SUPPLEMENTAL METHODS

Complete list of study's inclusion and exclusion criteria.

Inclusion criteria:

1. History of chronic symptomatic HF (ACC/AHA Class C) and New York Heart Association (NYHA) Class II or III symptoms at the time of enrollment.
2. Left ventricular ejection fraction (LVEF) of ≤45% assessed within 12 months prior to randomization by any imaging method.
3. Systolic blood pressure ≥90 mmHg.
4. Standard guideline-directed HF therapy.
5. If female of reproductive potential, agrees to avoid becoming pregnant while receiving study drug and for 14 days after the last dose of study drug by complying with abstinence from heterosexual activity or use (or have her partner use) contraception during heterosexual activity.

Exclusion criteria:

1. Addition of a new disease-modifying HF pharmacotherapy or CRT-D in previous 4 weeks.
2. Current or anticipated use of long-acting nitrates or nitric oxide (NO) donors including isosorbide dinitrate, isosorbide 5-mononitrate, pentaerythritol tetranitrate, nicorandil or transdermal nitroglycerin (NTG) patch, and molsidomine.
3. Current or anticipated use of phosphodiesterase type 5 (PDE5) inhibitors such as vardenafil, tadalafil, and sildenafil.

4. Current use or anticipated use of a soluble guanylate cyclase (sGC) stimulator such as riociguat.
5. Known allergy or sensitivity to any sGC stimulator.
6. Estimated glomerular filtration rate (eGFR) <15 mL/min/1.73 m2 or chronic dialysis.

   Patients who are pregnant or breastfeeding or plan to become pregnant or to breastfeed.

Brachial Artery Flow-Mediated Dilation Measurements and Analysis

Briefly, a BP cuff was positioned on the right forearm (below the antecubital crease) distal to the ultrasound Doppler probe. The brachial artery was insonated approximately midway between the antecubital and axillary regions, and measurements of brachial artery diameter and intensity-weighted mean blood velocity were acquired (Logiq 7, GE Medical Systems, Milwaukee, WI). After resting measurements of brachial artery diameter and mean blood velocity were performed, a blood pressure cuff was inflated to 250 mmHg for 5 min. After cuff release, data were recorded continuously for 2 minutes. Mean blood velocity was automatically calculated using commercially available software (Logiq 7). End-diastolic, electrocardiogram R-wave gated images were collected via video output from the Logiq 7 for off-line analysis of brachial artery diameter using automated edge-detection software (Medical Imaging Applications, Coralville, IA).

FMD was quantified as the maximal change in brachial artery diameter following cuff release, expressed as a percentage increase from pre-occlusion values (%FMD). The same investigators performed both the FMD measurements and analyses, and they were blinded to the measurement time points during the analysis. To maintain continuity between exams, investigators confirmed a similar distance between the antecubital fossa and the ultrasound probe on each study visit and ensured sample volume size and placement, as well as sampling frequency, were similar between exams.

Blood Biomarker Assays

Serum concentrations of N-Terminal Pro-Brain Natriuretic Peptide (NT-proBNP) were determined using a quantitative electrochemiluminescent immunoassay, high-sensitivity C-reactive protein (hsCRP) by quantitative immunoturbidimetry, and tumor necrosis factor-α (TNF-α) and interleukin-6 (IL-6) using a quantitative multiplex bead assay (ARUP Laboratories, Salt Lake City, UT). A commercially available enzyme-linked immunosorbent assay was used to analyze plasma concentrations of interleukin-18 (IL-18) (MBL Life Science, Japan).

Supplemental Table. Flow-mediated dilation measurements at baseline and week 12 by study group

	Placebo		Vericiguat		
	Baseline N = 12[1]	Week 12 N = 12[1]	Baseline N = 13[1]	Week 12 N = 13[1]	p-value[2]
Baseline BA diameter, mm	5.10 (0.82)	5.18 (0.92)	4.33 (0.78)	4.37 (0.72)	0.61
Peak BA diameter, mm	5.29 (0.83)	5.35 (0.86)	4.47 (0.79)	4.55 (0.76)	0.85
FMD / Shear rate AUC, %/ s^{-1}	0.12 (0.05)	0.12 (0.09)	0.11 (0.07)	0.11 (0.06)	0.89
Shear rate AUC, s^{-1}	36266.5 (22303.8)	32058.3 (15584.5)	32162.5 (13231.2)	38681.8 (13215.9)	0.08
Systolic blood pressure, mmHg	104.2 (16.4)	106.8 (13.2)	114.0 (13.5)	112.8 (16.5)	0.42
Diastolic blood pressure, mmHg	70.9 (11.1)	72.2 (10.0)	72.9 (10.7)	69.8 (7.5)	0.19

[1]Mean (SD)

[2]Two-way (group x time) ANOVA with repeated measures

BA: Brachial artery; FMD: Flow-mediated dilation; AUC: Area under the curve;

Supplemental Figure 1. Individual change for study outcomes between baseline and 12 weeks stratified by treatment group: **A)** flow-mediated dilation (FMD), **B)** N-terminal probrain natriuretic peptide (NT-proBNP), **C)** high-sensitivity C-reactive protein (hsCRP), **D)** interleukin 18 (IL-18), **E)** interleukin 6 (IL-6), **F)** tumor necrosis factor-α (TNF-α), **G)** six-minute walk test (6MWT), **H)** Kansas City Cardiomyopathy Questionnaire-12 overall summary score (KCCQ-12 OSS), **I)** visual analogue scale (VAS)

A)

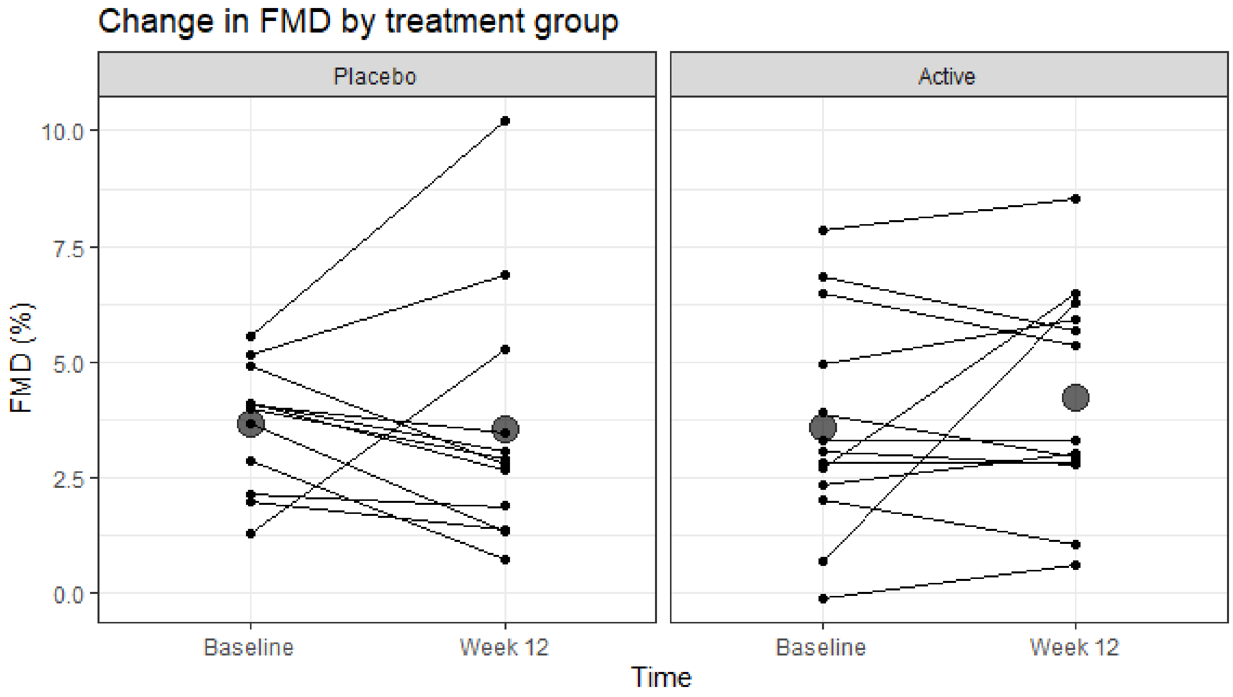

B)

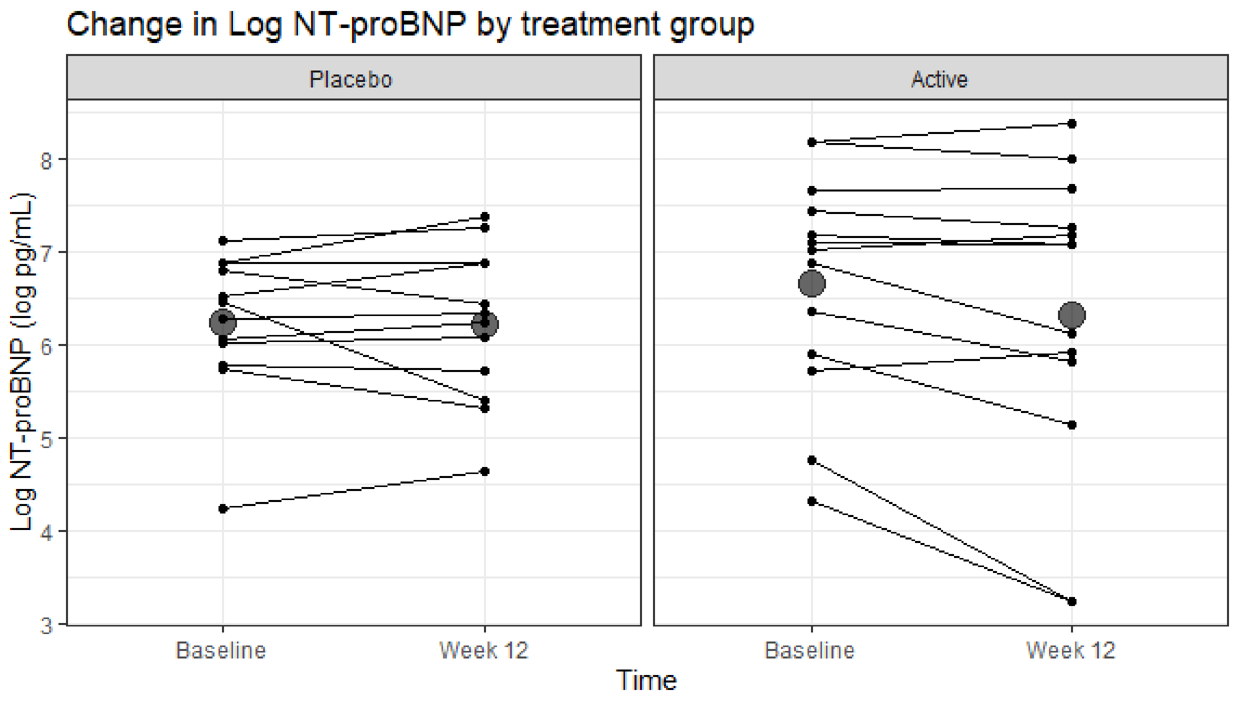
Change in Log NT-proBNP by treatment group
Placebo
Active
Log NT-proBNP (log pg/mL)
8
7
6
5
4
3
Baseline
Week 12
Baseline
Week 12
Time

C)

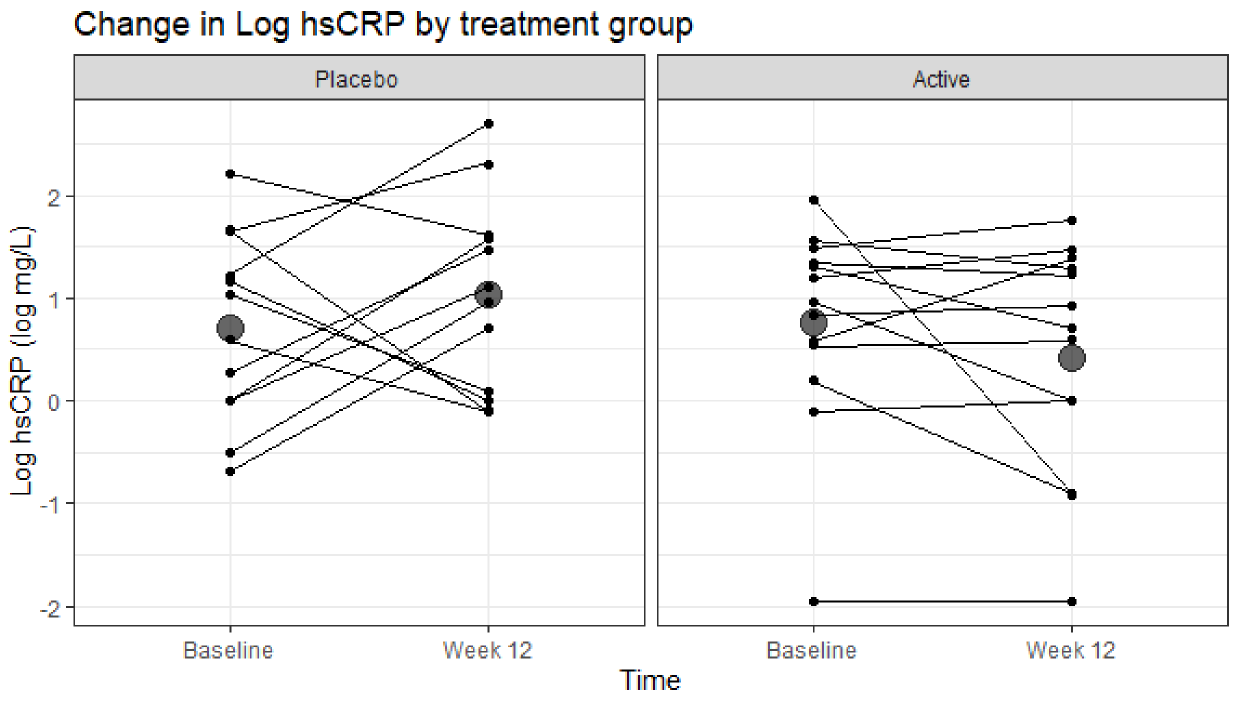

Change in Log hsCRP by treatment group
Placebo
Active
Log hsCRP (log mg/L)
2
1
0
-1
-2
Baseline
Week 12
Baseline
Week 12
Time

D)

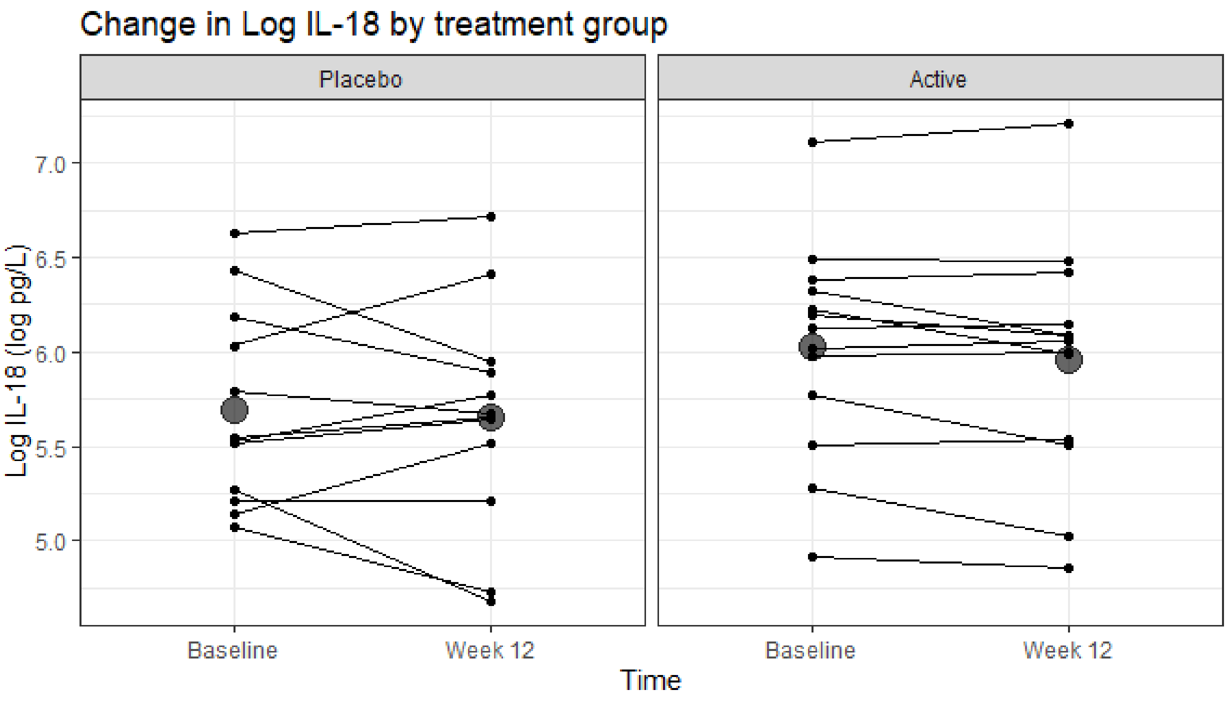
Change in Log IL-18 by treatment group
Placebo
Active
Log IL-18 (log pg/L)
7.0
6.5
6.0
5.5
5.0
Baseline
Week 12
Baseline
Week 12
Time

E)

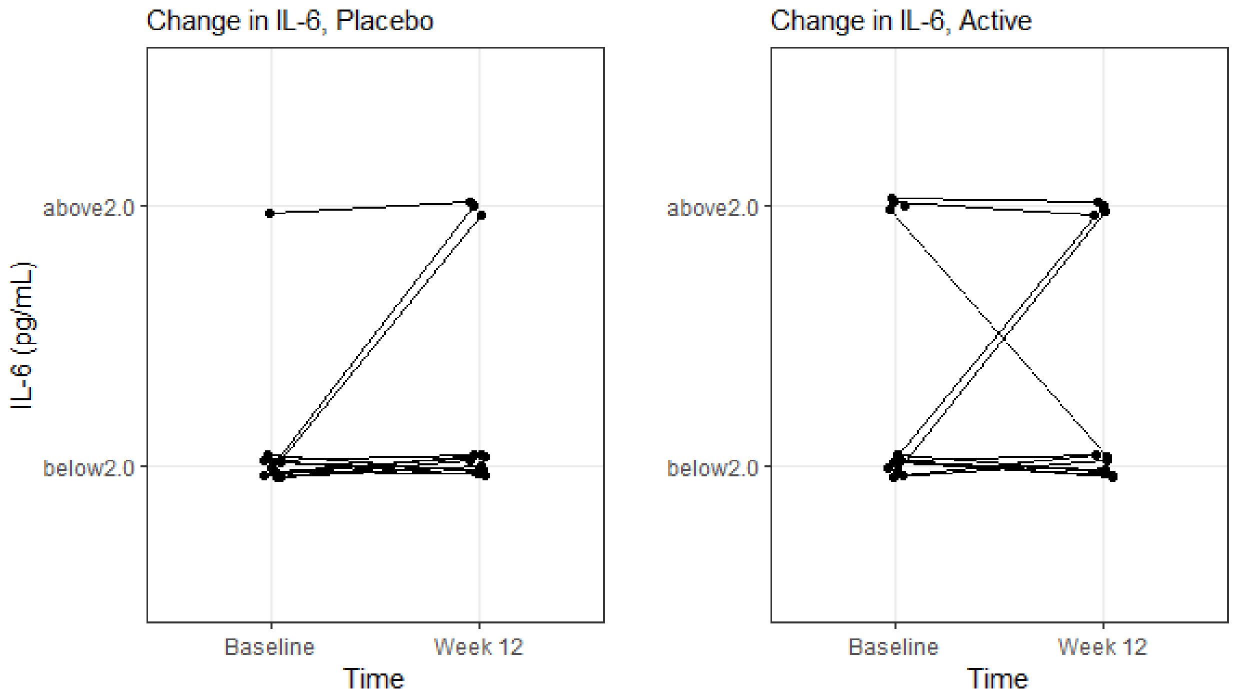

Change in IL-6, Placebo
Change in IL-6, Active
IL-6 (pg/mL)
above2.0
below2.0
Baseline
Week 12
Time

F)

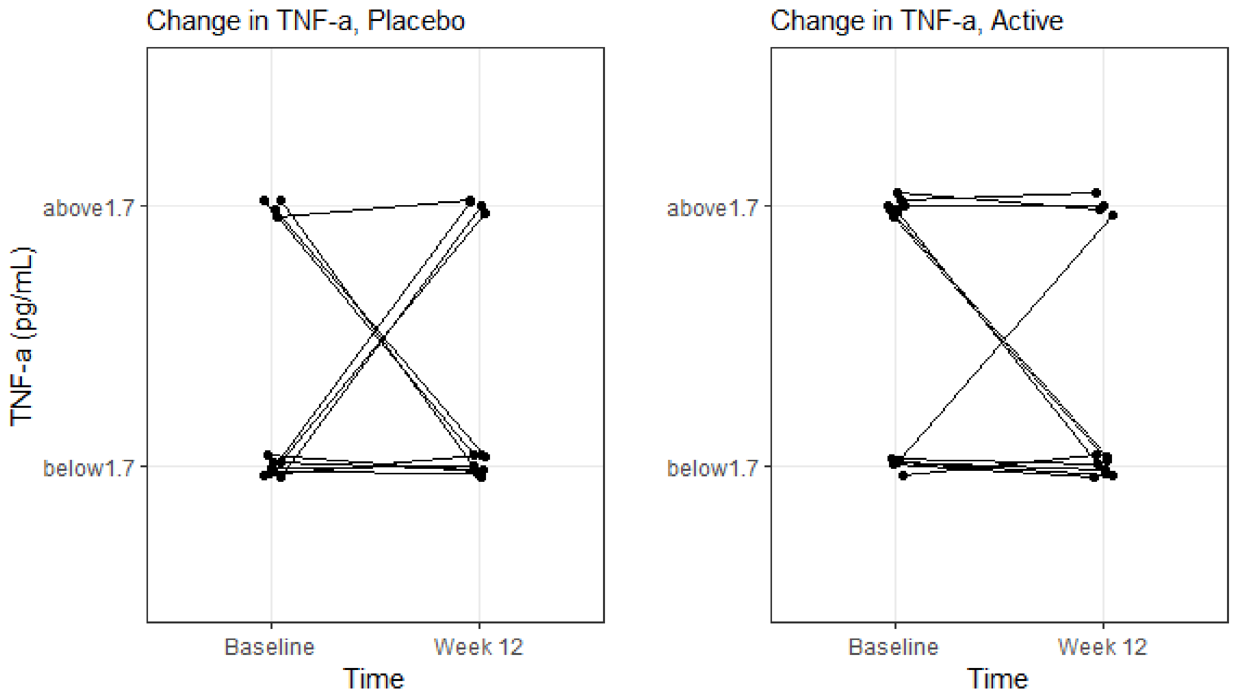
Change in TNF-a, Placebo
Change in TNF-a, Active
TNF-a (pg/mL)
above1.7
below1.7
Baseline
Week 12
Time

G)

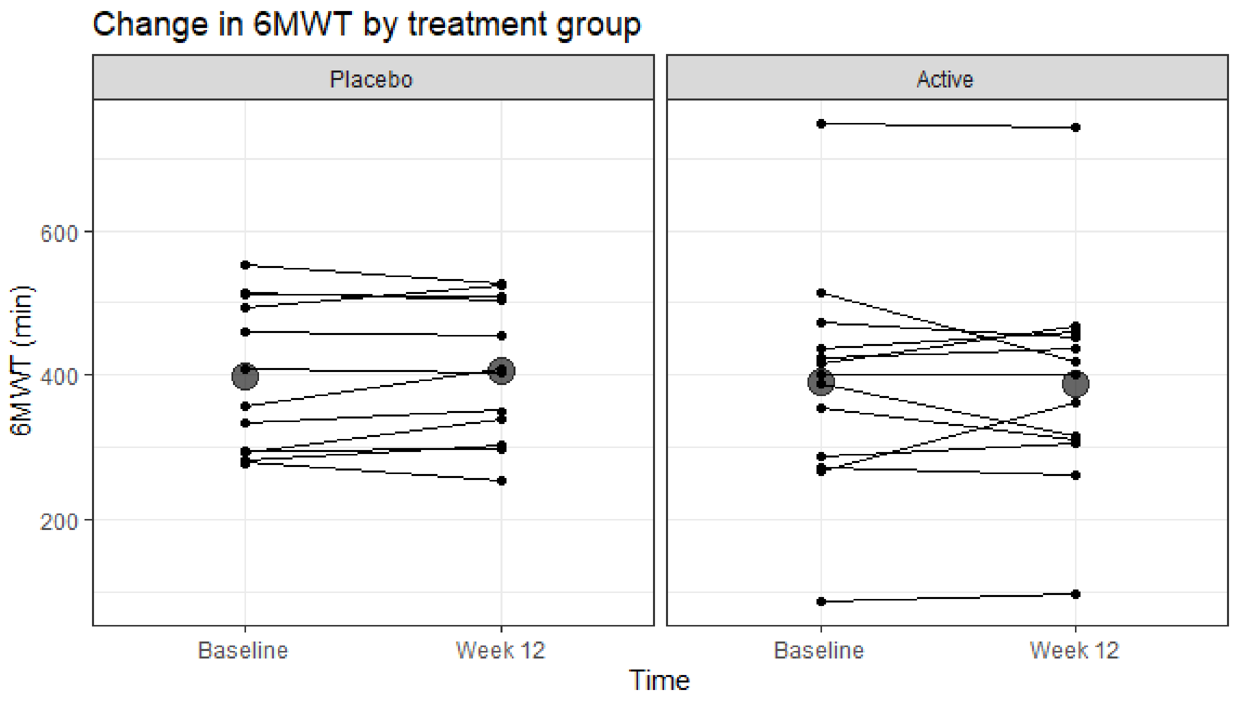

Change in 6MWT by treatment group
Placebo
Active
6MWT (min)
600
400
200
Baseline
Week 12
Baseline
Week 12
Time

H)

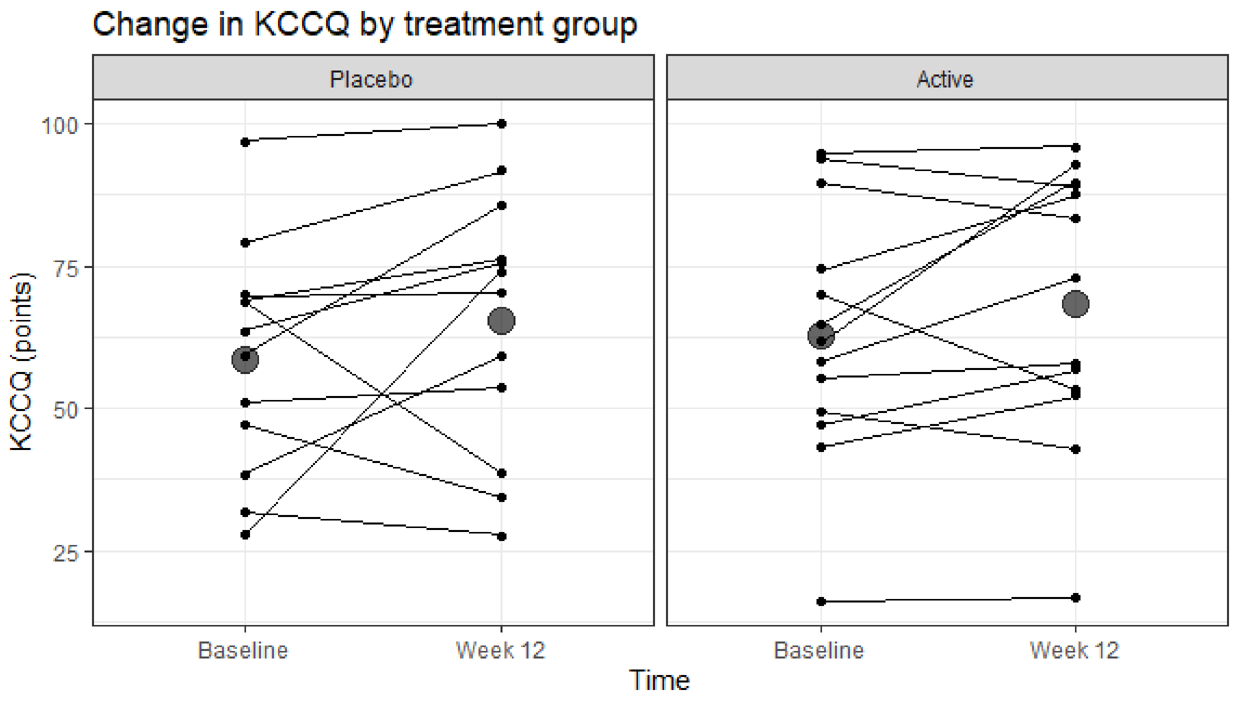

Change in KCCQ by treatment group
Placebo
Active
KCCQ (points)
100
75
50
25
Baseline
Week 12
Baseline
Week 12
Time

I)

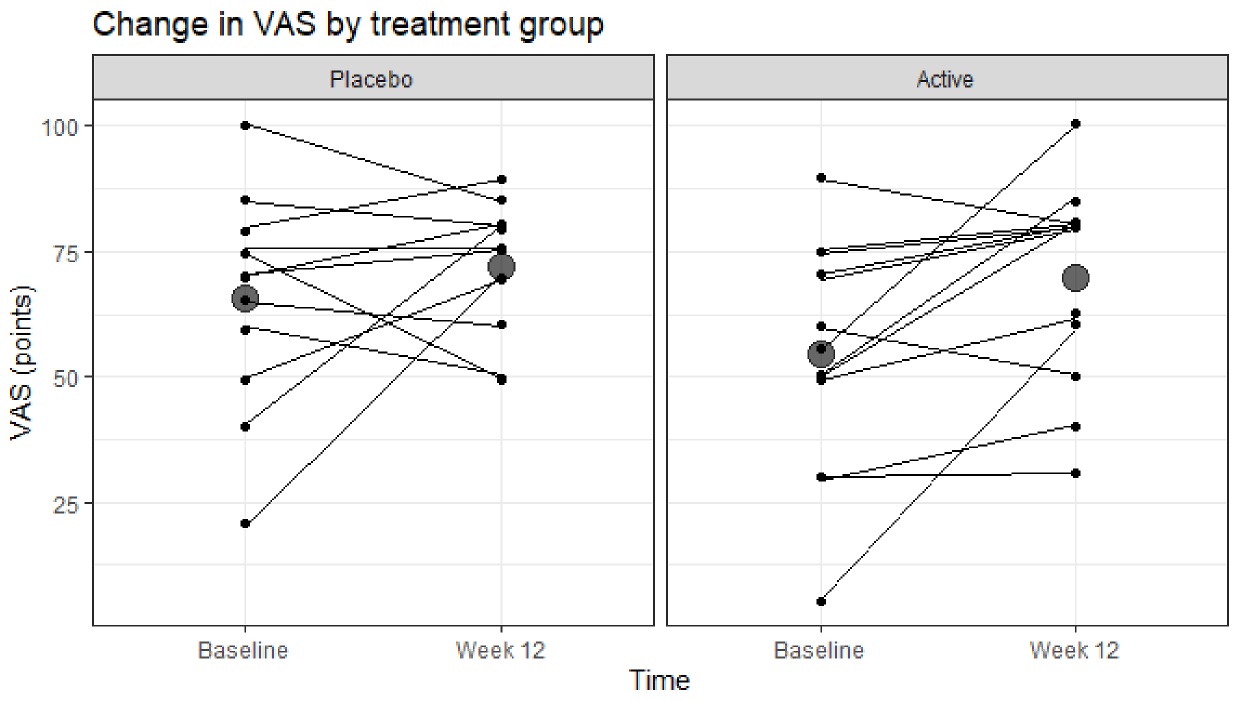

Change in VAS by treatment group
Placebo
Active
VAS (points)
100
75
50
25
Baseline
Week 12
Baseline
Week 12
Time
